# The Joint Analysis of Multi-Omics Data Revealed the Methylation-Expression Regulations in Atrial Fibrillation

**DOI:** 10.3389/fbioe.2020.00187

**Published:** 2020-03-12

**Authors:** Ban Liu, Xin Shi, Keke Ding, Mengwei Lv, Yongjun Qian, Shijie Zhu, Changfa Guo, Yangyang Zhang

**Affiliations:** ^1^Department of Cardiology, Shanghai Tenth People’s Hospital, Tongji University School of Medicine, Shanghai, China; ^2^Department of Pediatric Cardiovascular, Xin Hua Hospital, School of Medicine, Shanghai Jiao Tong University, Shanghai, China; ^3^Department of Cardiology, Shanghai Tongji Hospital, Tongji University School of Medicine, Shanghai, China; ^4^Shanghai East Hospital of Clinical Medical College, Nanjing Medical University, Shanghai, China; ^5^Department of Cardiovascular Surgery, Shanghai East Hospital, Tongji University School of Medicine, Shanghai, China; ^6^Department of Cardiovascular Surgery, National Clinical Research Center for Geriatric, West China Hospital, Sichuan University, Chengdu, China; ^7^Department of Cardiovascular Surgery, Zhongshan Hospital, Fudan University, Shanghai, China

**Keywords:** atrial fibrillation, methylation, multi-omics, feature selection, classification

## Abstract

Atrial fibrillation (AF) is one of the most prevalent heart rhythm disorder. The causes of AF include age, male sex, diabetes, hypertension, valve disease, and systolic/diastolic dysfunction. But on molecular level, its mechanisms are largely unknown. In this study, we collected 10 patients with persistent atrial fibrillation, 10 patients with paroxymal atrial fibrillation and 10 healthy individuals and did Methylation EPICBead Chip and RNA sequencing. By analyzing the methylation and gene expression data using machine learning based feature selection method Boruta, we identified the key genes that were strongly associated with AF and found their interconnections. The results suggested that the methylation of KIF15 may regulate the expression of *PSMC3*, *TINAG*, and *NUDT6*. The identified AF associated methylation-expression regulations may help understand the molecular mechanisms of AF from a multi-omics perspective.

## Introduction

Atrial fibrillation (AF), one of the most prevalent heart rhythm disorders, is a potential cause of heart failure and ischemic stroke with high morbidity and mortality (Ogawa et al., 2017; Asmarats et al., 2019). The cause of AF is multifactorial which include age, male sex, diabetes, hypertension, valve disease, and systolic/diastolic dysfunction (Schnabel et al., 2009; Lip et al., 2013; Voukalis et al., 2016). Depends on how often atrial fibrillation occurs and how it responds to treatment, AF is roughly divided into two major subtypes-paroxysmal atrial fibrillation (PAF) and persistent atrial fibrillation (PeAF). In the treatment of AF, drugs were the first choice, non-drug therapies were used only when drug therapy failed or patients could not tolerate the medication. In contrast to the extensive knowledge of etiology, the underlying mechanism of AF remains elusive. Further study of the potential mechanisms of AF could provide novel strategies for the treatment and management to increase quality of life and reduce economic burden of social (Chugh et al., 2001).

With the development of next generation sequencing (NGS) technologies, growing evidence have demonstrated that AF is a disease with a significant genetic contribution. Previous studies have filtered novel genetic variants and candidate genes including transcriptional factor genes (*PITX2, PRRX1, ZFHX3, NKX2.5*, *TBX5*), ion channel genes (*KCNN3*, *HCN4*, *CACNA1C*, *SCN5A*, *KCNQ1*, *KCNH2*), and caveolin genes (*CAV1* and *CAV2*) (Ellinor et al., 2010; Olesen et al., 2014; Sinner et al., 2014; Ma et al., 2016; Nielsen et al., 2018). However, these genes explain only a small fraction of the biology and genetic underpinnings of AF.

Epidemiological studies have demonstrated that genetic, environmental, behavioral, and clinical factors contribute to AF pathogenic mechanism (Zhong et al., 2016). Emelia J. B performed genome-wide methylation using whole blood samples from 183 prevalent AF and 220 incident AF cases. They examined the association between DNA methylation and GWAS loci, suggesting DNA methylation might be a possible mechanism through which AF-specific genetic variations affect gene regulation (Lin et al., 2017). To date, only a few studies have investigated differential DNA methylation as a predictor biomarker at specific candidate loci that were previously associated with AF.

Therefore, we applied DNA methylation profiling study to identify the likely rare damaging variants and putative candidate genes from 10 patients with persistent atrial fibrillation (PeAF), 10 patients with paroxymal atrial fibrillation (PAF) and 10 healthy individuals. Interestingly, we identified top 10 genes (KIF15, ABCA3, FOXG1, VGF, PDE4D, EIF3C, CNTNAP5, SHOX2, VGF, TRIM59) as functional candidate genes and the expression level are significantly increased in PeAF and PAF patients than control. Given the importance of DNA methylation to gene expression, we investigated the gene expression of the same participants using RNA sequencing. We also defined top 10 genes (*EPN3*, *EMD*, *SMCO4*, *F2RL2*, *TMED1*, *PSMC3*, *PDZD11*, *NUDT6*, *TINAG*, *GALNT5*) in gene expression data and the expression pattern of these genes was significantly different between PeAF and PAF. These results have improved our understanding of the underlying mechanism and offer new insights into the potential pathway of AF, which could provide novel therapeutic option for this disease.

## Materials and Methods

### Atrial Fibrillation Patients

Ten patients with paroxymal atrial fibrillation (g1), 10 patients with persistent atrial fibrillation (continuous atrial fibrillation lasting more than 12 months) (g2) and 10 healthy individuals (g3) were enrolled in this study (Table 1). All patients were subjected to detailed medical evaluation, which included medical history, physical examination, electrocardiography (ECG), and echocardiography. Patients with chronic heart failure, coronary heart disease, cardiomyopathy, hyperthyroidism or chronic pulmonary heart disease were excluded.

**TABLE 1:** Demographic characteristics of AF patients.

								Coronary			
	Age		Weight	Height			Diabetes	angiography	LVEF	Left atrial	Duration of
No.	(years)	Gender	(Kg)	(cm)	Smoking	Hypertension	mellitus	or CTA	(%)	diameter (mm)	AF (years)
1	69	Male	76	169	No	Yes	No	Negative	70	40	–
2	63	Male	64	170	No	No	No	Negative	59	46	–
3	63	Male	70	170	No	No	No	Negative	66	39	–
4	69	Male	67	173	No	Yes	No	Negative	67	46	–
5	69	Male	75	165	No	No	No	Negative	70	36	–
6	61	Male	76	176	No	Yes	Yes	Negative	60	42	–
7	64	Male	52	168	Yes	No	No	Negative	64	40	–
8	64	Male	71	181	No	Yes	Yes	Negative	63	39	–
9	61	Male	87	167	Yes	Yes	No	Negative	62	37	–
10	66	Male	82	173	No	Yes	No	Negative	63	42	–
11	63	Male	86	176	No	Yes	No	Negative	57	46	2.5
12	63	Male	80	178	No	No	No	Negative	68	55	3
13	64	Male	70	170	No	No	No	Negative	67	41	4
14	64	Male	84	164	No	Yes	No	Negative	55	48	2
15	65	Male	73	169	No	Yes	No	Negative	69	55	3.5
16	66	Male	66	168	No	Yes	No	Negative	64	45	4
17	67	Male	80	175	No	Yes	Yes	Negative	59	47	2.5
18	67	Male	73	165	Yes	Yes	No	Negative	59	47	3
19	63	Male	61	164	No	No	No	Negative	73	49	2
20	67	Male	90	178	No	Yes	No	Negative	70	58	2.5

*LVEF, left ventricular ejection fraction; AF, atrial fibrillation; CTA, CT angiography.*

The study was conducted in accordance with the Declaration of Helsinki, and the protocol used to collect human heart tissue was approved by the Ethics Committee of Shanghai East Hospital (DI: 0402017).

Written informed consents to participate in this study were provided by all the enrolled patients before operation of fibrillation ablation. The left atrial appendage tissues which were abandoned during isolated surgical ablation were collected. Normal left atrial appendages were collected from healthy male donors.

### The Methylation Profiles

The DNA methylation status of 850K probes in the 30 samples was measured using Methylation EPICBead Chip. The raw data was quality controlled and preprocessed using R/Bioconductor package minfi^1^ (Aryee et al., 2014). The beta value ranged from 0 to 1 was calculated to represent how each position was methylated. 1 meant high methylation and 0 meant low methylation.

### The RNA Sequencing Profiles

The total RNAs were extracted using RNeasy Mini Kit (Cat#74106, Qiagen) and the RNA integrity was checked using Agilent Bioanalyzer 2100 (Agilent technologies, Santa Clara, CA, United States). Qualified total RNA was further purified by RNAClean XP Kit (Cat A63987, Beckman Coulter Inc., Kraemer Boulevard, Brea, CA, United States) and RNase-Free DNase Set (Cat#79254, QIAGEN, GmBH, Germany). Pair-end sequencing reads were generated using Illumina data collection software. First, the reads were mapped onto human reference genome GRCh38 using Hisat2 (version:2.0.4^2^) (Kim et al., 2015). Then, Stringtie (version:1.3.0^3^) (Pertea et al., 2015) was used to calculate the FPKM (Fragments Per Kilobase of exon model per Million mapped reads).

### Feature Selection Algorithm

There were 866,091 methylation probes and 50,868 RNAs. The number of features were extremely large. It was difficult to select key features using traditional statistical methods. Therefore, we adopted the latest machine learning based feature selection method Boruta to get the key methylation probes and RNAs.

Boruta (Kursa and Rudnicki, 2010) is a feature selection method based on random forest. It can select sample group relevant features effectively. First, it will shuffle the features to create many permuted datasets. Then, it will evaluate the importance score of each feature in the original actual dataset and permuted datasets. Then, it will compare the actual importance score with permuted scores and find the features with significantly higher actual importance scores than permuted scores. After multiple iterations, it will select all the sample group relevant features. The python code from https://github.com/scikit-learn-contrib/boruta_py was used to apply the Boruta feature selection algorithm.

### Classification Predictor

To evaluate how well the selected features can classify the samples, we built an SVM (Support Vector Machine) classifier using the methylation data and another RNA-Seq data based SVM classifier. The svm function in R package e10171^4^ (Chang and Lin, 2011) was used to apply the SVM classification algorithm.

LOOCV (leave-one-out cross validation) was used to objectively evaluate the classification performance. Each time, one sample was treated as test sample while all the other samples were used to train the model. After 30 rounds, all samples had been tested once and the overall accuracy was calculated based on the confusion matrix. In confusion matrix, the actual sample groups were compared with predicted sample groups.

## Results

### The Key Methylation Features Identified With Boruta

We ran Boruta feature selection algorithm on the methylation data and got 10 key methylation features. These 10 key methylation features were listed in Table 2. The probes were annotated to genome positions (Genome Build 37) and genes using the official annotation file from Illumina. Sometime, one position may be associated with multiple genes. Therefore, the 10 methylation probes can be mapped onto 15 gene symbols. We checked the GO annotation of these genes and found that cg16703882 (*SHOX2*) was associated with GO:0007507: heart development which was closely relevant to AF.

**TABLE 2:** The 10 key methylation features identified by Boruta.

ILMNID	Chromosome	Position	Strand	UCSC Ref gene name
cg00702638	3	44803293	R	KIF15; KIAA1143
cg02331561	16	2391081	F	ABCA17P; ABCA3
cg02991338	14	29236017	R	FOXG1
cg04084157	7	100809049	F	VGF
cg05995159	5	59325256	R	PDE4D
cg06357615	16	28403195	R	MIR6862-2; MIR6862-1; EIF3CL; EIF3C
cg11344566	2	124782885	F	CNTNAP5
cg16703882	3	157823479	R	SHOX2
cg21186299	7	100808810	R	VGF
cg26856080	3	160167746	R	TRIM59

**FIGURE 1:**
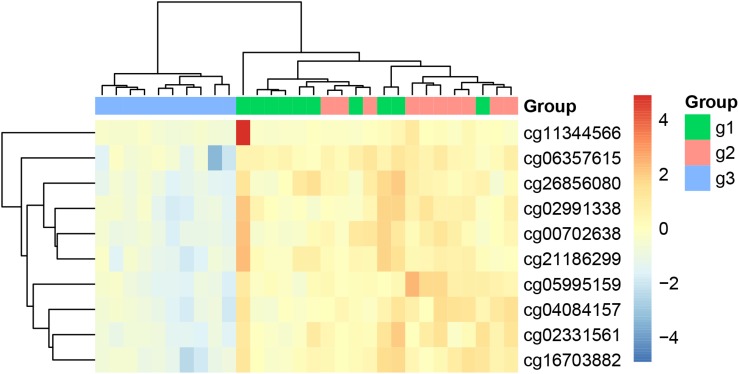
The heatmap of the key methylation features. The 30 samples were from three groups: 10 patients with paroxymal atrial fibrillation (g1), 10 patients with persistent atrial fibrillation (g2), and 10 healthy individuals (g3). It can be seen that most samples were cluster into the right groups.

We plotted the heatmap of these 10 key methylation features in Figure 1. It can be seen that most of the 10 patients with paroxymal atrial fibrillation (g1), 10 patients with persistent atrial fibrillation (g2) and 10 healthy individuals (g3) were cluster into the right groups.

### The Key Gene Expression Features Identified With Boruta

**FIGURE 2:**
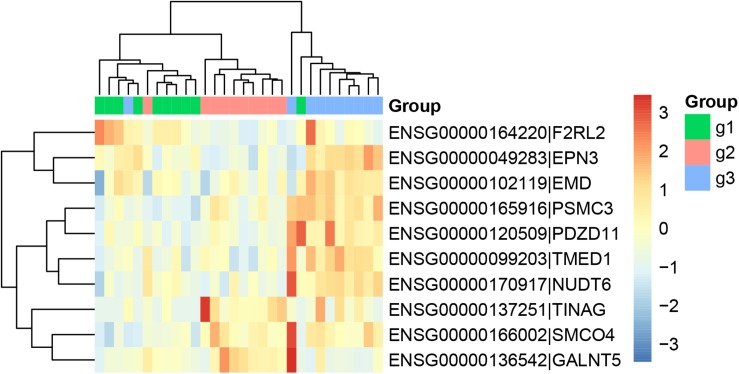
The heatmap of the key gene expression features. The 30 samples were from three groups: 10 patients with paroxymal atrial fibrillation (g1), 10 patients with persistent atrial fibrillation (g2), and 10 healthy individuals (g3). It can be seen that most samples were cluster into the right groups.

Similarly, we ran Boruta feature selection algorithm on the RNA-Seq gene expression data and got 10 key gene expression features. The 10 key gene expression features were given in Table 3. We also plotted their heatmap in Figure 2. The clusters were also largely correct.

**TABLE 3:** The 10 key gene expression features identified by Boruta.

Gene ID	Name	Description	GRCh38 locus
ENSG00000049283	EPN3	Epsin 3	17:50532543-50543750
ENSG00000102119	EMD	Emerin	X:154379197-154381523
ENSG00000166002	SMCO4	Single-pass membrane protein with coiled-coil domains 4	11:93478472-93543508
ENSG00000164220	F2RL2	Coagulation factor II (thrombin) receptor-like 2	5:76615482-76623434
ENSG00000099203	TMED1	Transmembrane p24 trafficking protein 1	19:10832438-10836318
ENSG00000165916	PSMC3	Proteasome 26S subunit, atpase 3	11:47418769-47426473
ENSG00000120509	PDZD11	PDZ domain containing 11	X:70286595-70290514
ENSG00000170917	NUDT6	Nudix hydrolase 6	4:122888697-122922968
ENSG00000137251	TINAG	Tubulointerstitial nephritis antigen	6:54307859-54390152
ENSG00000136542	GALNT5	Polypeptide N-acetylgalactosaminyltransferase 5	2:157257598-157314211

### The Classification Performance of Methylation and Gene Expression

From Figures 1, 2, we can see that both methylation and gene expression features can correctly cluster most samples. But we would like to evaluate their performance objectively and quantitatively. Therefore, we applied LOOCV to test the SVM classifiers of methylation and gene expression features. The confusion matrixes of methylation features and gene expression features were listed in Tables 4, 5, respectively.

**TABLE 4:** The confusion matrix of key methylation features.

	Predicted g1	Predicted g2	Predicted g3
Actual g1	8	2	0
Actual g2	3	7	0
Actual g3	0	0	10

**TABLE 5:** The confusion matrix of key gene expression features.

	Predicted g1	Predicted g2	Predicted g3
Actual g1	9	0	1
Actual g2	0	9	1
Actual g3	1	0	9

From Table 3, we can see that the AF patients (g1 + g2) and healthy individuals (g3) were perfectly classified using the methylation features, but the methylation data did not have great performance on classifying the subtype of AF (g1 and g2). From Table 4, we can see that the two subtype of AF, paroxysmal atrial fibrillation (g1) and persistent atrial fibrillation (g2), had very different gene expression pattern. In other words, the methylation data and gene expression data complement each other. The methylation data can be used to predict the AF and the gene expression data can be used to classify the subtypes of AF.

We checked the wrongly predicted samples in Tables 3, 4. They were different. Within the 30 samples, 22 samples had the same predicted labels by expression and methylation. All these 22 samples were correctly predicted. For the 8 inconsistent samples between expression and methylation predictions, at least one of the two predictions (the expression-based prediction and the methylation-based prediction) was correct. In other words, all the samples can be corrected classified based on either expression or methylation. The expression-based prediction and the methylation-based prediction were complementary.

### The Methylation-Expression Regulation Network

**FIGURE 3:**
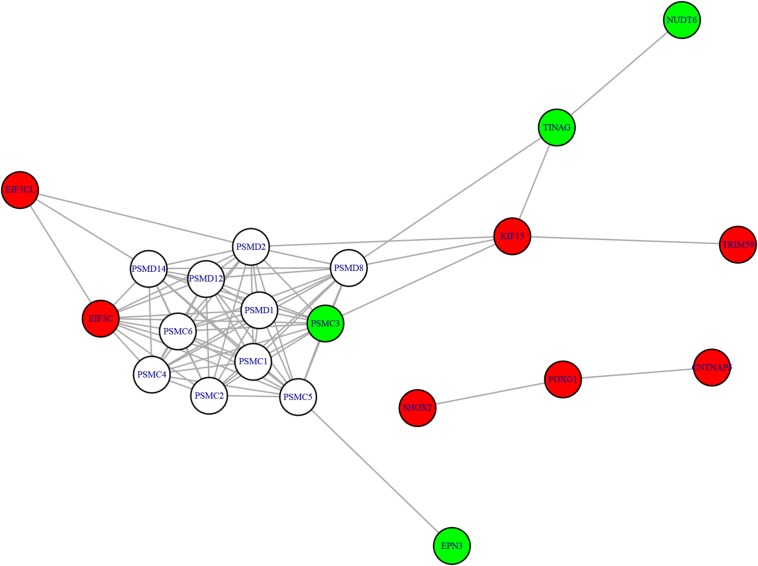
The methylation-expression regulation network. The red and green nodes were methylation and expression genes, respectively. The methylation genes located in three clusters: EIF3CL-EIF3C, KIF15-TRIM59, and SHOX2-FOXG1-CNTNAP5. KIF15 can directly or indirectly regulate the expression genes and may play important roles.

We mapped the genes of methylation features and gene expression features to the STRING network (Version 11.0^5^) (Szklarczyk et al., 2018) and visualized the network using R package igraph (Csardi and Nepusz, 2006)^6^ to identify the potential relationship between two candidate genes sets. The methylation-expression regulation network was shown in Figure 3. In the network, the methylation and expression genes were marked in red and green. The methylation genes located in three clusters: *EIF3CL-EIF3C*, *KIF15-TRIM59*, and *SHOX2-FOXG1-CNTNAP5*. The three expression genes (*PSMC3*, *TINAG*, and *NUDT6*) were connected with methylation gene *KIF15*. Even *EPN3* can be indirectly connected to *KIF15*. That made *KIF15* at the center of the network. These results suggested that *KIF5* may play important roles in the pathogenesis of AF.

## Discussion

DNA methylation, a pre-transcriptional modification characterized by the addition of methyl groups to specific nucleotides, regulates the stability of gene expression states and maintains genome integrity by collaborating with proteins that modify nucleosomes (Ma et al., 2014; Tao et al., 2016; Shen et al., 2017). Previous studies considered that changes in DNA methylation states contribute to the regulation of biological processes underlying AF, such as fibrosis, atrial dilatation, atrial fibroblast proliferation and differentiation from fibroblasts into myofibroblasts (Zhao et al., 2017). To further enhance the biological understanding of the atrial fibrillation, our study focused on DNA methylation, particularly with respect to how it relates to mRNA expression. Among our two gene sets of top 10 genes, we found PDED4, SHOX2, and EMD were the most important genes for AF which have been reported associated with AF in previous reference.

Atrial fibrillation is reported to be associated with a profound remodeling of membrane receptors and alterations in cAMP dependent regulation of Ca2^+^ handling. PDE4 is expressed in human atrial myocytes and accounts for approximately 15% of PDE (phosphodiesterase) activity (Molina et al., 2012). PDE4D encoded protein has 3’,5’-cyclic-AMP phosphodiesterase activity and degrades cAMP, which acts as a signal transduction molecule in multiple cell types and represents the major PDE4 subtype (Berk et al., 2016). The activity of PDEs decreased with age, and the relative PDED4 activity was lower in patients with permanent atrial fibrillation than in age-matched sinus rhythm controls (Milton et al., 2011). Previous study provided evidence that patients with pAF were found to have a decreased PDE4 activity as compared with patients in sinus rhythm (Yeh et al., 2007).

Short Stature Homeobox 2 (*SHOX2*) is a member of the homeobox family of genes in which mutations associated with early-onset and familial AF (Hoffmann et al., 2019). *SHOX2* is considered as a key regulator of sinus node development of which deficiency could lead to bradycardia in animal models (Vicente-Steijn et al., 2017). Previous study demonstrated *SHOX2* was susceptible for SND and AF by screening 98 SND patients and 450 individuals with AF. In the heart development of mouse and zebrafish, they also proved *SHOX2* plays an important role, the mutation of SHOX2 could lead to severe bradycardia (Blaschke et al., 2007; Ye et al., 2015).

Emerin (EMD) encodes a serine-rich nuclear membrane protein which located on the cytoplasmic surface of the inner nuclear membrane and related to X-linked Emery-Dreifuss muscular dystrophy (EDMD) (Capanni et al., 2009). Previous study found a nonsense mutation in EMD from two EDMD families which is associated with X-linked recessive inheritance, result in serious cardiac complication, including AF (Sakata et al., 2005). Cardiologic assessment revealed slow atrial fibrillation in a recent case of a 65-year-old male patient with a hemizygous duplication of 5 bases in exon 6 of the EMD, gene on the X chromosome (Kissel et al., 2009; Zhao et al., 2014; Brisset et al., 2019).

## Data Availability Statement

The raw data supporting the conclusions of this article will be made available by the authors, without undue reservation, to any qualified researcher.

## Ethics Statement

The studies involving human participants were reviewed and approved by the Ethics Committee of Shanghai East Hospital. The patients/participants provided their written informed consent to participate in this study. Written informed consent was obtained from the individual(s) for the publication of any potentially identifiable images or data included in this manuscript.

## Author Contributions

YZ and CG conceived and designed the experiments. BL, XS, and KD performed the experiments. ML, YQ, and SZ analyzed the data. BL, XS, and YZ wrote the manuscript. All authors read and approved the final version of the manuscript.

## Conflict of Interest

The authors declare that the research was conducted in the absence of any commercial or financial relationships that could be construed as a potential conflict of interest.

## Footnotes

1 https://bioconductor.org/packages/minfi/

2 http://ccb.jhu.edu/software/hisat2/

3 http://ccb.jhu.edu/software/stringtie/

4 https://CRAN.R-project.org/package=e1071

5 http://string-db.org

6 https://CRAN.R-project.org/package=igraph
